# Interaction between insulin and androgen signalling in decidualization, cell migration and trophoblast invasion *in vitro*

**DOI:** 10.1111/jcmm.16892

**Published:** 2021-08-31

**Authors:** Angelica Lindén Hirschberg, Ivika Jakson, Carlota Graells Brugalla, Daniel Salamon, Dorina Ujvari

**Affiliations:** ^1^ Department of Women’s and Children’s Health Karolinska Institutet Stockholm Sweden; ^2^ Department of Gynecology and Reproductive Medicine Karolinska University Hospital Stockholm Sweden; ^3^ Department of Microbiology, Tumor and Cell Biology Karolinska Institutet Stockholm Sweden

**Keywords:** androgens, decidualization, endometrium, insulin, interaction

## Abstract

Finely tuned decidualization of endometrial stromal fibroblasts into decidual cells is crucial for successful implantation and a healthy pregnancy. Both insulin and androgens are known to modulate decidualization, however, their complex effect on this process has not been fully elucidated. As hyperinsulinemia and hyperandrogenism are associated in clinical conditions, we aimed to investigate the interaction between insulin and androgens on decidualization. Primary human endometrial stromal cells were decidualized *in vitro* in the presence of insulin and/or androgens (dihydrotestosterone (DHT), testosterone). Gene or protein expressions of decidualization markers were measured, and cells size characteristics were determined. Migration of decidualizing endometrial stromal cells and invasion of HTR‐8/SVneo trophoblast spheroids were assessed. We found that insulin and androgens in combination enhanced the upregulation of several decidualization markers including prolactin, tissue factor, tissue inhibitor of matrix metalloproteinase 3 and connexin‐43, and also interacted in modulating cell size characteristics resulting in enlarged decidualizing cells. However, insulin and DHT together restricted the migration of decidualizing cells and invasion of trophoblast spheroids. Our findings suggest that insulin and androgens interact to potentiate the process of decidualization. On the other hand, inhibited cell migration and trophoblast invasion might negatively impact the function of decidualizing endometrial stromal cells.

## 1 INTRODUCTION

Decidualization denotes the morphological and biochemical differentiation of endometrial stromal cells in the secretory phase of the menstrual cycle. During decidualization, spindle‐shaped fibroblast‐like endometrial stromal cells enlarge and acquire epithelioid characteristics, which is associated with enlarged nucleus, increased number of nucleoli, accumulation of glycogen and lipid droplets, dilatation of the rough endoplasmic reticulum and the Golgi systems and increased number of gap junctions between the neighbouring cells.^1^, ^2^ This transformation is of importance for embryo implantation and the formation of a functional feto‐maternal interface as it controls trophoblast proliferation, migration and invasion.^3^ A variety of factors are secreted from decidualized cells and have been proposed to serve as putative markers of decidualization, including prolactin (PRL), insulin‐like growth factor binding protein 1 (IGFBP1), tissue factor (TF), tissue inhibitor of matrix metalloproteinase 3 (TIMP3), prokineticin 1 (PROK1) and the main gap junction protein connexin‐43 (CX43).^4^ Impaired decidualization is considered to be associated with reproductive disorders, including decreased implantation, recurrent miscarriage and placenta‐related disorders.^5^

The role of insulin in decidualization has been investigated in several studies, however, its effect on decidualization markers has shown varying results. Thus, it has been demonstrated that insulin downregulates IGFBP1 in decidualizing human endometrial stromal cells,^6^, ^7^, ^8^ whereas PRL production is stimulated.^9^ We have furthermore reported that insulin downregulates a number of decidualization markers via the transcriptional inactivation of Forkhead box protein 1 (Foxo1), a crucial transcription factor in decidualization,^8^ whereas PROK1 is highly enhanced by insulin in decidualizing human endometrial stromal cells.^10^ However, it was recently shown that adequate insulin signalling via insulin receptor substrate‐2 supports the decidualization process.^7^

Androgens may also play a role for decidualization.^11^, ^12^, ^13^ In the endometrium, the androgen receptor (AR) expression is confined to the stroma and it fluctuates during the menstrual cycle with a gradual decrease from the early proliferative to the mid‐secretory phase.^14^ Furthermore, endometrial expression levels of enzymes that play a role in the biosynthesis and conversion of androgens are higher in the secretory phase of the menstrual cycle, suggesting a role of locally synthesized androgens in decidualization.^14^, ^15^ It was recently demonstrated that androgens (testosterone and dihydrotestosterone (DHT)) enhance the expression of decidualization markers such as PRL and IGFBP1 and promote the morphological and ultrastructural changes associated with the decidualization process.^1^, ^15^, ^16^

Hyperinsulinemia and hyperandrogenism are common clinical conditions in polycystic ovary syndrome (PCOS) and obesity. These conditions are related to implantation failure, increased risk of miscarriage and adverse pregnancy outcomes.^17^, ^18^, ^19^, ^20^ However, the mechanisms by which hyperinsulinemia and/or hyperandrogenism weaken the chance for a successful implantation and an uncomplicated pregnancy are still not elucidated. The purpose of the present study was to investigate the *in vitro* interaction between insulin and androgens on the decidualization process, focusing on the functional changes in decidualizing endometrial stromal cells.

## 2 MATERIALS AND METHODS

### 2.1 Subjects

Regularly cycling, healthy volunteers (*n* = 9) underwent collection of endometrial biopsy under local anaesthesia using a suction curette (Pipet Curet, CooperSurgical) in the proliferative phase of the menstrual cycle at cycle day 5–9. All women were between 18 and 35 years and had a body mass index between 19 and 28. Exclusion criteria were hormonal medication within 3 months prior to biopsy sampling, smoking, endocrine disorder, current chronic disease or continuous medication. They gave their written informed consent and the Regional Ethical Committee in Stockholm approved the study (DNR 2018/2199‐31).

### 2.2 Isolation of human endometrial stromal cells and culture conditions

Isolation of human endometrial stromal cells was carried out as described previously.^8^ Endometrial stromal cells were seeded to 6‐well Costar plates (Sigma‐Aldrich) at a density of 10^5^/well and cultured in DMEM/F12‐Glutamax medium (Thermo Fischer Scientific) supplemented with 10% heat‐inactivated foetal bovine serum (HI‐FBS; Sigma‐Aldrich) and 0.2% penicillin‐streptomycin (Sigma‐Aldrich) until 80%–90% confluency. *In vitro* decidualization of endometrial stromal cells was induced in phenol red‐free DMEM/F12 (Thermo Fischer Scientific) supplemented with 2% charcoal‐stripped FBS (Sigma‐Aldrich) 0.2% penicillin‐streptomycin (Sigma‐Aldrich) using 1 μM medroxyprogesterone‐17‐acetate (MPA; Sigma‐Aldrich) and 0.5 mM dibutyryl‐cAMP (db‐cAMP; Sigma‐Aldrich) in the presence or absence of 100 nM insulin (Sigma‐Aldrich), 1 μM DHT (Sigma‐Aldrich) or 1 μM testosterone (Sigma‐Aldrich), or the combination of insulin and DHT or insulin and testosterone for six days. The culture media was renewed after 3 days.

In the experiments of flow cytometry, wound‐healing assay and spheroid co‐culture invasion assay, we chose to treat the cells with insulin and/or DHT, but not with testosterone, since DHT has the strongest affinity to the androgen receptor^21^ and does not convert to other hormones.

### 2.3 RNA isolation, cDNA synthesis and RT‐PCR

Total RNA was extracted using Quick‐RNA Miniprep Kit (Zymo Research) and subjected to cDNA synthesis using SuperScript VILO cDNA Synthesis Kit (Thermo Fischer Scientific). Gene expression levels of PRL and IGFBP1 were determined with TaqMan method. TF and TIMP3 were measured using the SybrGreen method. Ribosomal protein L13A (RPL13A) was used as a housekeeping gene to normalize gene expression levels. Gene expression levels were analysed with the ΔΔC_t_ method. The employed TaqMan assays (Thermo Fischer Scientific) and oligonucleotides (Sigma‐Aldrich) are listed in Tables S1 and S2. All determinations were performed in triplicate.

### 2.4 Flow cytometry

Flow cytometry was used to determine protein expression of CX43. Cell cultures were harvested with 3 ml AutoMACS Rinsing solution (Miltenyi Biotec, Germany) at 37°C and pelleted with centrifugation at 400 g for 7 min. Cells were resuspended in 180 μl Cytofix/Cytoperm (BD Biosciences), transferred to V shaped 96‐well plate (Sigma‐Aldrich) and incubated for 20 min on ice. After centrifugation at 450 g for 6 min, cells were washed/permeabilized twice using 180 μl Perm/Wash buffer (BD Biosciences), and after centrifugation, cells were resuspended in 40 μl Perm/Wash buffer. Five microlitre Fc block (BD Biosciences) was applied for 15 min at room temperature in order to block non‐specific binding of potential Fc receptors. Cells were then incubated with APC‐conjugated CX43 or isotype control antibody (R&D Systems) for 1 h at 4°C in dark, washed twice using 180 μl Perm/Wash buffer and resuspended in 130 μl fixation buffer (BioLegend) prior to flow cytometric analysis. The fluorescence of the cells was measured using a NovoCyte flow cytometer (ACEA Biosciences, Inc.), and data were analysed with the FlowJo software (TreeStar).

We also determined forward scatter (FSC—reflecting cell volume), side scatter (SSC—reflecting the granularity or the internal complexity of the cells) and pulse‐width (reflecting cell diameter) parameters in order to approximate cell size.

### 2.5 Cell size determination using light microscopy

After 5 days of decidualization, cells were detached from the culture dishes using TrypLE Express (Thermo Fischer Scientific) and microphotographs were taken with 100x magnification using a Leica DFC420 C digital camera on a Nikon Eclipse TS 100 inverted microscope. At least 100 cells were analysed in each condition of each healthy donor. Photographs were analysed using ImageJ software.

### 2.6 Wound‐healing assay

A wound‐healing assay was used to study the migratory potential of decidualized cells after treatment with insulin, DHT or the combination of both. Endometrial stromal cells were seeded in 24‐well Costar plates (Sigma‐Aldrich) and cultured in DMEM/F12‐Glutamax supplemented with 10% HI‐FBS and 0.2% penicillin‐streptomycin until confluency. The cells were decidualized as stated above in the presence or absence of 100 nM insulin, 1 μM DHT or their combination for 6 days. The cells were scratched with a 200 μl pipette tip and washed twice with PBS. 700 μl phenol red‐free DMEM/F12 media supplemented with 2% charcoal‐stripped foetal bovine serum and 0.2% penicillin‐streptomycin was added to each well. No decidualization agents, insulin or DHT were added during the 24 h of the wound‐healing assay. The migration of the cells was followed by an IncuCyte S3 Live‐Cell Analysis System (Sartorius) using a 4x objective (whole well, phase contrast imaging) for 24 h. Photographs were analysed using ImageJ software. The experiments were performed with cells from five healthy volunteers. Each experiment was performed in duplicate.

### 2.7 Formation of trophoblast spheroids and co‐culture invasion assay

We applied a co‐culture invasion assay to investigate the invasion of decidualized cells by HTR‐8/SVneo spheroids in the presence of insulin, DHT or their combination. Spheroids consisting of 3*10^3^ HTR‐8/SVneo, a first trimester derived immortalized trophoblast cell line, were formed as described previously.^10^ The co‐culture experiments were performed in 24‐well Costar plates. Primary endometrial stromal cells were seeded at a density of 10^5^/well and cultured until 90% confluency. Cells were either decidualized in the presence or absence of 100 nM insulin, 1 μM DHT or their combination as described above for 6 days or left untreated. Then, media was changed to 700 μl phenol red‐free DMEM/F12 supplemented with 2% charcoal‐stripped FBS and 0.2% penicillin‐streptomycin. One HTR‐8/SVneo spheroid was carefully transferred to each well onto the confluent stromal/decidual cells using 1 ml pipette tip previously cut with sterile blade in order to widen it. No decidualization agents, insulin or DHT were added during the 16 h of the co‐culture invasion assay. The invasion of HTR‐8/SVneo spheroids was followed by an IncuCyte S3 Live‐Cell Analysis System using a 4x objective (whole well, phase contrast imaging). The invasion areas of spheroids were measured using ImageJ. The experiments were performed with cells from five healthy volunteers.

### 2.8 Statistical analysis

Statistical analysis was performed with GraphPad Prism 9.0. Two‐way ANOVA for repeated measurements was used with the within group factors insulin (yes/no), DHT (yes/no) or testosterone (yes/no) and the interaction insulin*DHT or insulin*testosterone. Considering the limited sample size and the high risk of type II error (false‐negative results), we performed simple main effect tests if interactions corresponded to *p* < 0.15. All the data were log‐transformed because of skewness. *p* < 0.05 was considered statistically significant.

## 3 RESULTS

### 3.1 Gene expression levels of decidualization markers in response to insulin, androgens and combined treatment

*In vitro* decidualization increased all studied markers (Figure S1A–D).

### 3.2 PRL

**FIGURE 1:**
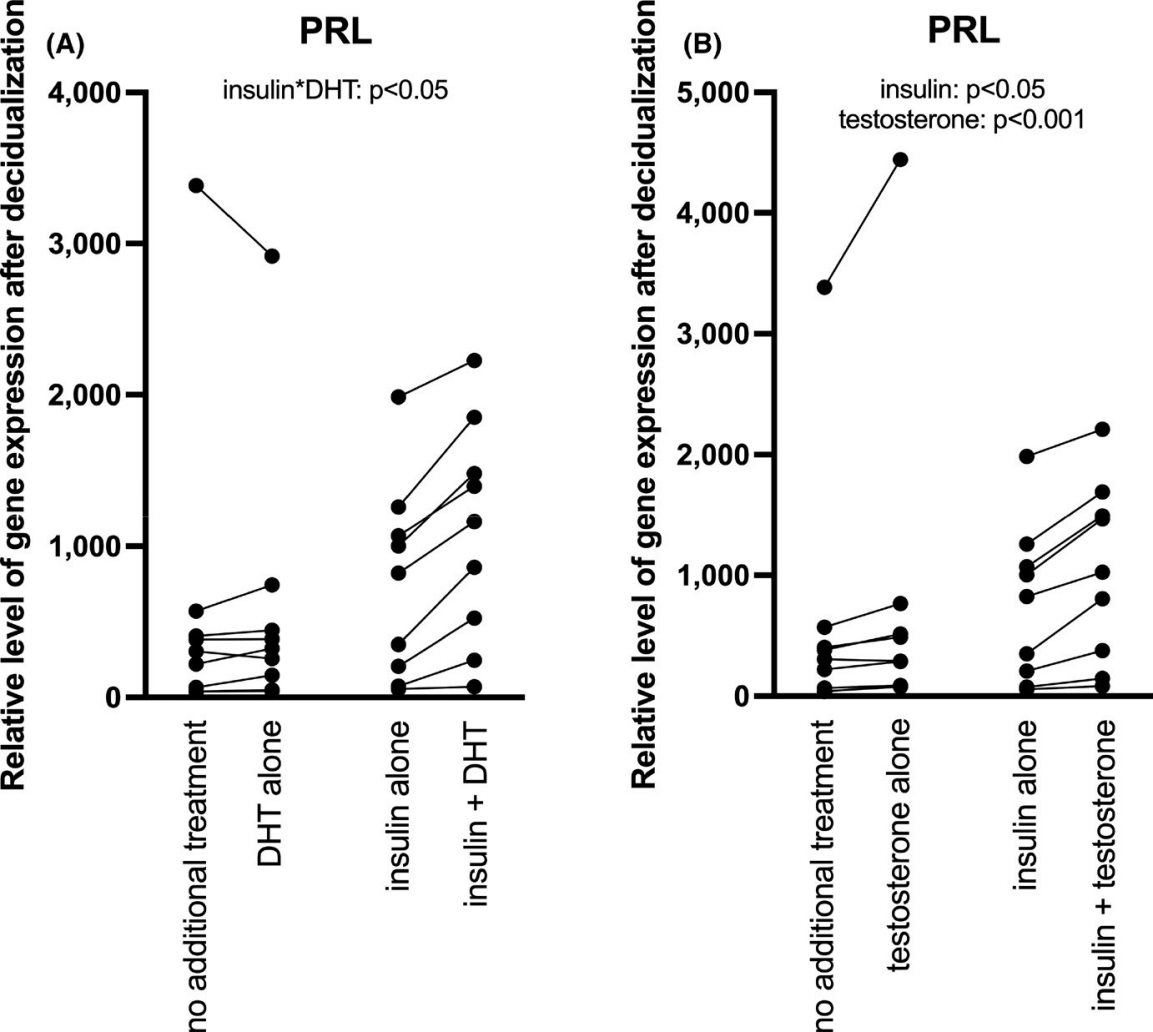
(A) Relative levels of PRL gene expression after 6 days in response to insulin and/or DHT in *in vitro* decidualized (treated with MPA and db‐cAMP) human endometrial stromal cells of healthy volunteers. Interaction effect between insulin and DHT: *p* < 0.05. (B) Relative levels of PRL gene expression after 6 days in response to insulin and/or testosterone in *in vitro* decidualized (treated with MPA and db‐cAMP) human endometrial stromal cells of healthy volunteers. Main effect of insulin: *p* < 0.05; main effect of testosterone: *p* < 0.001

The interaction between insulin and DHT was significant, and the post hoc test showed higher PRL expression by insulin in combination with DHT than for insulin (*p* = 0.003) or DHT alone (*p* = 0.002; Figure 1 and Table 1). Furthermore, insulin increased PRL expression compared with no treatment (*p* = 0.038). There was no interaction between insulin and testosterone, but a significant main effect for both insulin and testosterone (*p* = 0.030 and *p* = 0.000, respectively (Figure 1 and Table 1).

**TABLE 1:** Decidualization markers in *in vitro* decidualized endometrial stromal cells in response to insulin, DHT or testosterone and insulin+DHT or insulin+testosterone

	Gene	Decidual	Insulin	Androgen	Insulin+Androgen	Interaction and main effect	Post‐hoc effects
DHT	PRL	306 (39–3386)	823 (56–1986)	326 (44–2917)	1164 (71–2228)	1^(*)^	a^(*)^, b^(**)^, d^(**)^
	IGFBP1	13,436 (191–277,298)	13,805 (28–303,133)	20,800 (373–220,117)	19,510 (161–547,130)		
	TF	2.22 (0.62–39.04)	5.82 (1.37–248.00)	3.01 (0.92–71.60)	4.60 (1.35–220.50)	1^(*)^	a^(***)^, b^(**)^, c^(**)^
	TIMP3	2.41 (1.33–9.63)	16.74 (2.38–69.46)	4.08 (2.13–19.62)	36.47 (3.39–121.5)	A^(**)^, B^(*)^	
	CX43	1.8x10^5^ (1.4x10^5^‐3.2x10^5^)	2.5x10^5^ (1.7x10^5^‐3.8x10^5^)	1.7x10^5^ (1.2x10^5^‐3.0x10^5^)	2.5x10^5^ (1.7x10^5^‐4.0x10^5^)	1^(*)^	a^(**)^, b^(**)^
Testosterone	PRL	306 (39–3386)	823 (56–1986)	289 (79–4444)	1025 (82–2209)	A^(*)^, B^(***)^	
	IGFBP1	13,436 (191–277,298)	13,805 (28–303,133)	14,889 (262–238,187)	21,879 (128–462,294)	B^(**)^	
	TF	2.22 (0.62–39.04)	5.82 (1.37–248.00)	2.56 (0.85–82.37)	7.10 (1.48–307.50)	A^(****)^, B^(**)^	
	TIMP3	2.41 (1.33–9.63)	16.74 (2.38–69.46)	4.83 (2.16–9.57)	19.47 (3.75–65.77)	A^(**)^, B^(*)^	

Note

Data are median and ranges (min‐max). Values of PRL, IGFBP1, TF and TIMP3 are relative gene expression levels compared with those in stromal cells. Values of CX43 are protein expression levels (AU (arbitrary unit) of mean fluorescence intensity). 1 = Interaction between insulin and androgen, A = Main effect of insulin, B = Main effect of androgen. Post hoc test: a = insulin vs. decidual, b = insulin+androgen vs. androgen, c = androgen vs. decidual, d = insulin+androgen vs. insulin. *p < 0.05, **p < 0.01, ***p < 0.001 and ****p < 0.0001.

### 3.3 IGFBP1

There was no interaction or significant main effects of insulin or DHT on IGFBP1 gene expression (Table 1 and Figure S2). Furthermore, there was no significant interaction between insulin and testosterone. However, testosterone increased IGFBP1 (*p* = 0.005) but not insulin (Table 1 and Figure S2).

### 3.4 TF

There was an interaction between insulin and DHT, and the post hoc test showed higher TF expression by the combined insulin and DHT treatment compared with DHT alone (*p* = 0.005). Furthermore, both insulin and DHT increased TF expression compared with no treatment (*p* = 0.000 and *p* = 0.004, respectively; Table 1 and Figure S2). There was no interaction between insulin and testosterone, but both insulin and testosterone increased TF (*p* = 0.000 and *p* = 0.006, respectively; Table 1 and Figure S2).

### 3.5 TIMP3

There was no significant interaction between insulin and DHT, however, both increased the gene expression of TIMP3 independently of each other (*p* = 0.001 and *p* = 0.016, respectively; Table 1 and Figure S2). The effect of testosterone exposure was similar to that of DHT treatment (Table 1 and Figure S2).

### 3.6 Protein expression levels of the gap junction protein CX43 in response to insulin, DHT and their combined treatment

*In vitro* decidualization did not significantly affect CX43 protein expression (not shown). There was an interaction between insulin and DHT, and the post hoc test showed a significantly higher protein expression of CX43 when cells were treated with insulin and DHT in combination compared with DHT alone (*p* = 0.002). Insulin alone, also increased CX43 compared with no treatment (*p* = 0.007; Table 1 and Figure S3).

### 3.7 Cell size determination

The interaction between insulin and DHT was significant for most parameters of cell characteristics (Table 2). Insulin in combination with DHT increased FSC‐A (reflecting cell volume) compared with insulin alone (*p* = 0.001), but not insulin or DHT alone (Table 2 and Figure S4). Furthermore, the combined treatment increased SSC‐A (reflecting cell complexity) compared with DHT alone (*p* = 0.000), and insulin alone increased SSC‐A compared with no treatment (*p* = 0.005; Table 2 and Figure S4). Insulin in combination with DHT also increased pulse‐width (reflecting cell diameter) compared with insulin and DHT alone (*p* = 0.0058 and *p* = 0.0067, respectively; Table 2 and Figure S4). Similarly, insulin in combination with DHT increased cell size compared with insulin and DHT alone (*p* = 0.002 and *p* = 0.020, respectively; Table 2 and Figure S4).

**TABLE 2:** Cell characteristics of *in vitro* decidualized endometrial stromal cells in response to insulin, DHT and insulin+DHT

Measure	Decidual	Insulin	DHT	Insulin+DHT	Interaction and main effect	Post hoc effects
FSC‐A	6.0 x 10^5^ (2.6 x 10^5^−7.5 x 10^5^)	5.6 × 10^5^ (2.9 × 10^5^−8.0 × 10^5^)	5.4 × 10^5^ (2.3 × 10^5^−8.3 × 10^5^)	6.0 × 10^5^ (3.4 × 10^5^−8.6 × 10^5^)	1^(*)^	d^(**)^
SSC‐A	8.7 × 10^5^ (4.6 × 10^5^−1.4 × 10^6^)	1.2 × 10^6^ (7.9 × 10^5^−1.6 × 10^6^)	8.3 × 10^5^ (4.0 × 10^5^−1.4 × 10^6^)	1.3 × 10^6^ (7.3 × 10^5^−1.7 × 10^6^)	1^(*)^	a^(**)^, b^(***)^
Pulse width	113.0 (89.0–149.0)	121.0 (101.0–144.0)	114.0 (87.0–149.0)	121.0 (107.0–152.0)	1^(^ *^p^* ^=0.058)^	b^(**)^, d^(**)^
Cell size	2468 (1817–2833)	3184 (2492–3388)	2257 (1794–2586)	3456 (2719–3652)	1^(*)^	b^(*)^, d^(**)^

Note

Data are median and ranges (min‐max). The units of FSC‐A, SSC‐A and pulse width are AU (arbitrary unit) of mean fluorescence intensity. The units of cell size are pixel^2^. 1 = Interaction between insulin and DHT. Post hoc test: a = insulin vs. decidual, b = insulin+DHT vs. DHT, c = DHT vs. decidual, d = insulin+DHT vs. insulin. *p < 0.05, **p < 0.01 and ***p < 0.001.

### 3.8 Wound‐healing assay

**FIGURE 2:**
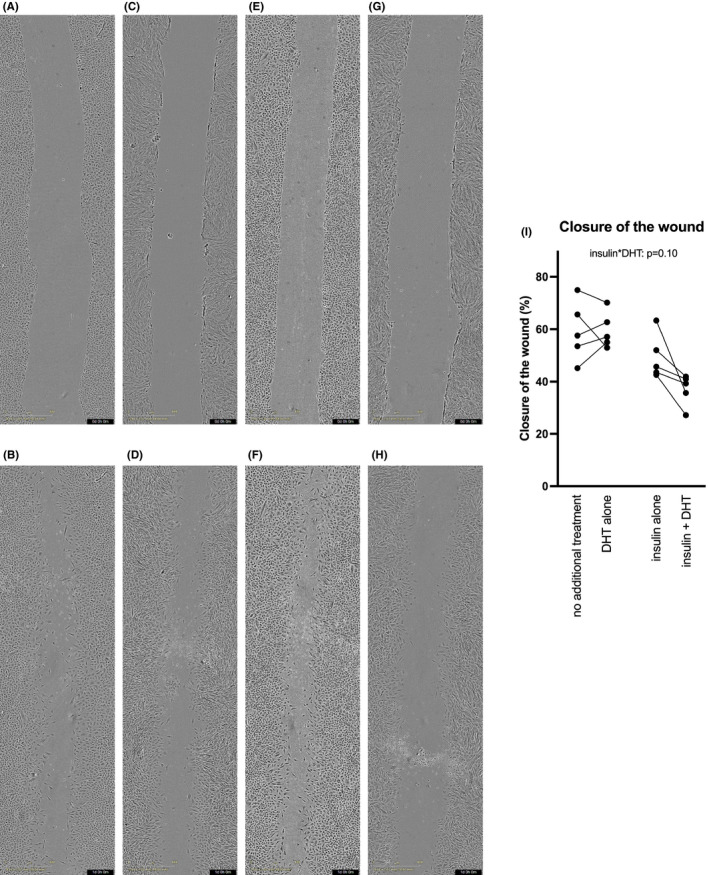
(A–H) Representative microphotographs (40x magnification) of wound‐healing assay of stromal cells decidualized (treated with MPA and db‐cAMP) for 6 days after wounding at 0h (A) and 24h (B), with insulin treatment at 0h (C) and 24h (D), with DHT treatment at 0h (E) and 24h (F) and with insulin and DHT treatment at 0h (G) and 24h (H) were taken with IncuCyte S3 Live‐Cell Analysis System. (I) Closure of the wound using *in vitro* decidualized (treated with MPA and db‐cAMP) human endometrial stromal cells of healthy volunteers in response to insulin and/or DHT after 6 days, at 24 h. Interaction effect between insulin and DHT: *p* = 0.10

I*n vitro* decidualization did not significantly affect the migration of the cells as compared to undifferentiated stromal cells (not shown). Insulin and DHT interacted in cell migration of decidualizing cells, and post hoc test showed that the combined treatment inhibited cell migration in comparison with insulin or DHT alone (*p* = 0.045 and *p* = 0.005, respectively; Figure 2A–I and Table S3).

### 3.9 Co‐culture invasion assay

**FIGURE 3:**
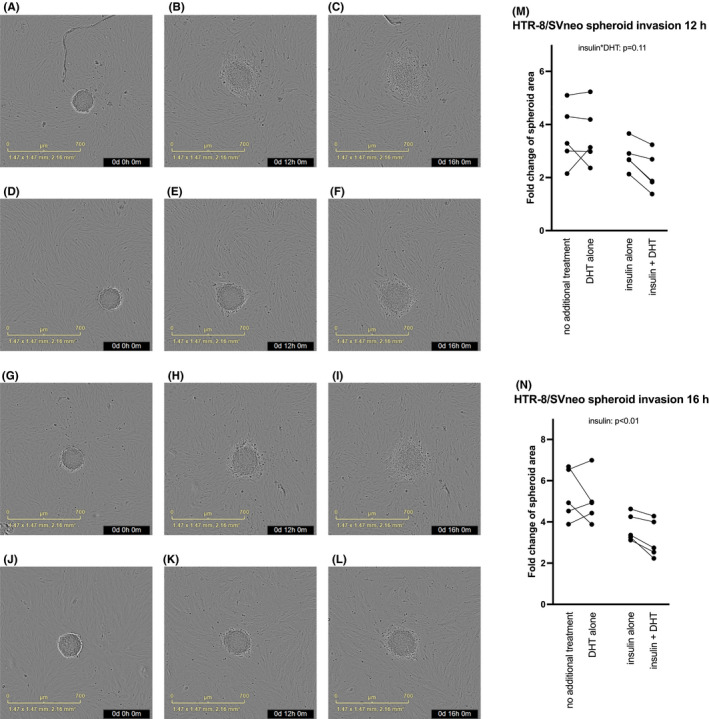
(A‐L) Representative microphotographs (40x magnification) of co‐culture invasion assay using HTR‐8/SVneo spheroids and stromal cells decidualized (treated with MPA and db‐cAMP) for 6 days at 0h (A), 12h (B) and 16h (C), with insulin treatment at 0h (D), 12h (E) and 16h (F), with DHT treatment at 0h (G), 12h (H) and 16h (I), and with insulin and DHT treatment at 0h (J), 12h (K) and 16h (L) were taken with IncuCyte S3 Live‐Cell Analysis System. (M) Co‐culture invasion assay using HTR‐8/SVneo spheroids on *in vitro* decidualized (treated with MPA and db‐cAMP) human endometrial stromal cells of healthy volunteers in response to insulin and/or DHT after 6 days, at 12h. Interaction effect between insulin and DHT: *p* = 0.11. (N) Co‐culture invasion assay using HTR‐8/SVneo spheroids on *in vitro* decidualized (treated with MPA and db‐cAMP) human endometrial stromal cells of healthy volunteers in response to insulin and/or DHT after 6 days, at 16h. Main effect of insulin: *p* < 0.01

*In vitro* decidualization did not significantly affect the invasion of the HTR‐8/SVneo spheroids when compared to that of undifferentiated stromal cells (not shown). Insulin and DHT interacted in invasion of trophoblast spheroids of decidualizing cells, and post hoc test demonstrated that the combined treatment with insulin and DHT inhibited the outgrowth of HTR‐8/SVneo trophoblast spheroids at 12 h compared with both insulin and DHT alone (*p* = 0.019 and *p* = 0.010, respectively). The effect of the combined treatment of insulin and DHT was similar at 16 h. Furthermore, at 16 h, insulin significantly restricted spheroid outgrowth (*p* = 0.001; Figure 3A–N, Videos [Link], [Link], [Link], [Link] and Table S3).

## 4 DISCUSSION

This is the first study demonstrating evidence of interaction between insulin and androgens in the regulation of decidualizing endometrial stromal cells. The upregulation of several decidualization markers and the increase of decidual cell size were enhanced by insulin, and also by androgens to a lesser extent, whereas the combination of insulin and DHT seemed to potentiate this effect even further. However, insulin and particularly the combined treatment of insulin and DHT inhibited migration of decidualizing cells and invasion of trophoblast spheroids.

Our results concerning the separate effects of insulin and androgens on the decidualization markers PRL and IGFBP1 are largely consistent with earlier data. Thus, it was previously shown that both intracrine and exogenous androgens enhance the production of PRL and IGFBP1 along with the morphological transformation into decidualizing endometrial cells.^1^, ^15^, ^16^, ^22^ On the other hand, previous studies have demonstrated that insulin enhances PRL and PROK1 productions, whereas IGFBP1 is downregulated in decidualizing endometrial stromal cells.^6^, ^7^, ^8^, ^9^, ^10^

Here, we found that both insulin and androgens enhanced PRL, and testosterone also increased IGFBP1, whereas insulin had no significant effect on IGFBP1. In addition, we have shown that the decidualization markers TF and TIMP3 were upregulated by insulin and androgens. In these experiments, we tested both DHT and testosterone, however, since there were no major differences in the actions of these two androgens on decidualization markers, we chose to use only DHT in the following experiments. This was because DHT has a 2‐fold higher affinity to the androgen receptor than testosterone.^21^ Furthermore, testosterone can exert diverse effects due to its conversion to both oestrogen and DHT in decidualizing endometrial stromal cells, via the action of aromatase and 5α‐reductase type 1, respectively.^22^

The main findings in our study are the interactions between insulin and androgens in the regulation of decidualization, showing a potentiated upregulation of gene expression of PRL and TF and of protein expression of the gap junction protein CX43 by insulin and DHT in combination. There were also significant interactions in the modulation of cell size characteristics, with the greatest increase in decidualizing endometrial cell size by the combination of insulin and DHT. The mechanisms of these interactions are not known. However, hypothetically it may involve the phosphatidylinositol 3‐kinase (PI3K)‐Akt (protein kinase B) signalling pathway. In peripheral tissues, insulin and IGF‐I signalling involves activation of the PI3K‐Akt pathway. Several modes of interaction between Akt and androgen signalling via AR have been suggested, however, predominantly in prostate cancer, including direct phosphorylation and inhibition of AR by Akt, regulation by Akt via the wnt/GSK3B/ß‐catenin pathway, crosstalk of AR and Akt involving NF‐ kB and suppression of AR by Foxo1.^23^, ^24^, ^25^ It remains to be determined whether insulin and androgens interact in decidualizing endometrial stromal cells via an action of PI3K/Akt signalling and which downstream pathways are involved in this process.

Successful invasion of the embryonic trophoblasts requires sufficient motility of maternal endometrial stromal cells.^26^, ^27^, ^28^, ^29^, ^30^ It has been demonstrated that stromal cells migrate away from the implanting blastocyst, but also move around and encapsulate the early embryo, thereby facilitating the invasion of trophoblasts into the decidual bed.^26^, ^29^, ^31^ However, there needs to be a delicate balance between mechanisms that promote trophoblast invasion and those that restrict it. Inadequate implantation is considered to increase the risk of early miscarriage and placental insufficiency, whereas excessive trophoblast invasion might lead to placenta accrete.^32^

Several putative factors are involved in the regulation of endometrial stromal cell migration and trophoblast invasion including hormones, growth factors, chemokines and inflammatory mediators.^33^ Wongwananuruk and collaborators studied the role of androgen signalling and demonstrated that DHT alone had no significant effect on trophoblast invasion in a co‐culture system using HTR‐8/SVneo trophoblast cells and decidualizing endometrial stromal cells.^31^ We found that combined treatment with insulin and DHT inhibited migration of decidualizing stromal cells, whereas there was no significant effect of insulin or DHT alone. Furthermore, the combined treatment, as well as insulin alone, restricted the invasion of trophoblast spheroids.

The decreased migration and trophoblast invasion by the combined treatment could at least partly be explained by the increased expression of gap junction protein CX43. However, even though we washed the decidualizing stromal cells several times when performing the wound‐healing and co‐culture assays, it cannot be excluded that the effect of decidual secretion of other factors, such as PROK1 might have impacted the migration and invasion. We have previously demonstrated that PROK1, a crucial negative regulator of trophoblast invasion,^10^, ^34^, ^35^ also decreases the migration of endometrial stromal cells^10^ and is enhanced by the combined treatment with insulin and androgens.^36^ Taken together, our results show that interaction of insulin and androgens causes decreased endometrial stromal cell migration along with increased expression of CX43, and inhibited trophoblast invasion *in vitro*, that suggest dysregulation of the decidualization process.

There are certain limitations with our study. We used extravillous trophoblast cell line HTR‐8/SVneo instead of primary cells and the number of endometrial samples are limited in the study. However, we consider the use of primary endometrial stromal cells instead of cell lines as a strength of the study.

In conclusion, our results demonstrate that insulin and androgens interact in the decidualization process, leading to enlarged decidualizing stromal cells and enhanced upregulation of several putative decidualization markers. However, at the same time the combined treatment of insulin and androgens caused inhibition of endometrial stromal cell migration and trophoblast invasion *in vitro*. We suggest that these results may indicate a dysregulation of the normally finely tuned decidualization. The results may have implications for the understanding of implantation failure, miscarriage and placenta‐related pregnancy complications in conditions of hyperinsulinemia and hyperandrogenism, such as PCOS and obesity.

## CONFLICT OF INTEREST

The authors declare no conflicts of interest.

## AUTHOR CONTRIBUTION

**Angelica Lindén Hirschberg:** Conceptualization (lead); Data curation (lead); Formal analysis (lead); Funding acquisition (lead); Investigation (lead); Methodology (equal); Project administration (lead); Resources (lead); Supervision (lead); Validation (lead); Writing – original draft (lead); Writing – review and editing (equal). **Ivika Jakson:** Conceptualization (equal); Data curation (equal); Formal analysis (equal); Investigation (equal); Methodology (equal); Writing – review and editing (equal). **Carlota Graells Brugalla:** Data curation (equal); Formal analysis (equal); Investigation (equal); Writing – review and editing (equal). **Daniel Salamon:** Data curation (equal); Formal analysis (equal); Investigation (equal); Methodology (equal); Writing – review and editing (equal). **Dorina Ujvari:** Conceptualization (lead); Formal analysis (lead); Funding acquisition (lead); Investigation (lead); Methodology (lead); Project administration (lead); Resources (lead); Supervision (lead); Validation (lead); Visualization (lead); Writing – original draft (lead); Writing – review and editing (equal).

## Supporting information

Fig S1

Fig S2

Fig S3

Fig S4

Table S1

Table S2

Table S3

Video S1

Video S2

Video S3

Video S4

Supplementary Material

## Data Availability

The data that support the findings are available from the corresponding author upon reasonable request.
