# Supplementary figures and images for: Interaction between insulin and androgen signalling in decidualization, cell migration and trophoblast invasion in vitro
Source: J Cell Mol Med. 2021 Aug 31;25(20):9523–32. doi: 10.1111/jcmm.16892 (PMC8505820; doi:10.1111/jcmm.16892)

Supplementary figure 1

A B C D


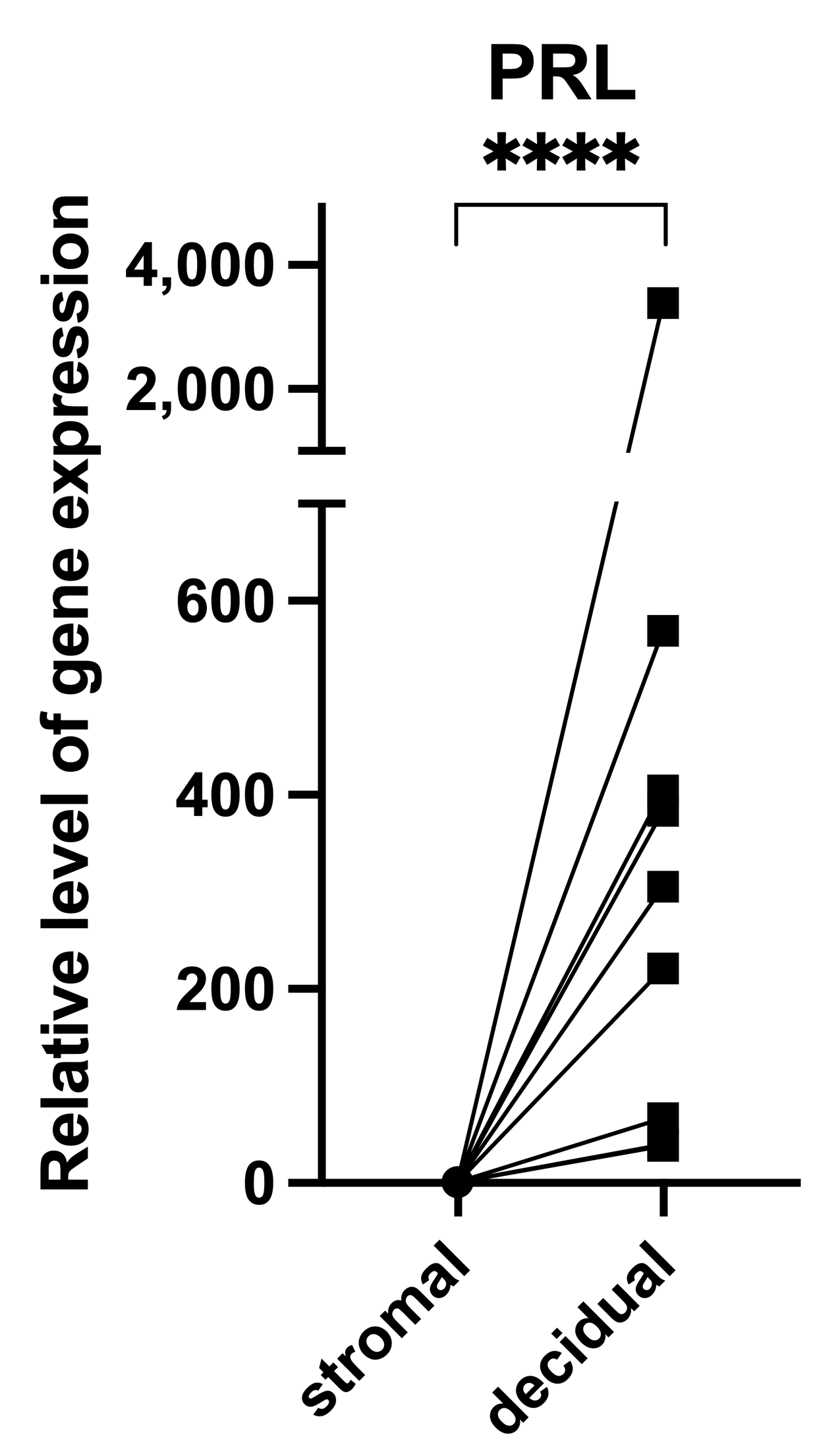

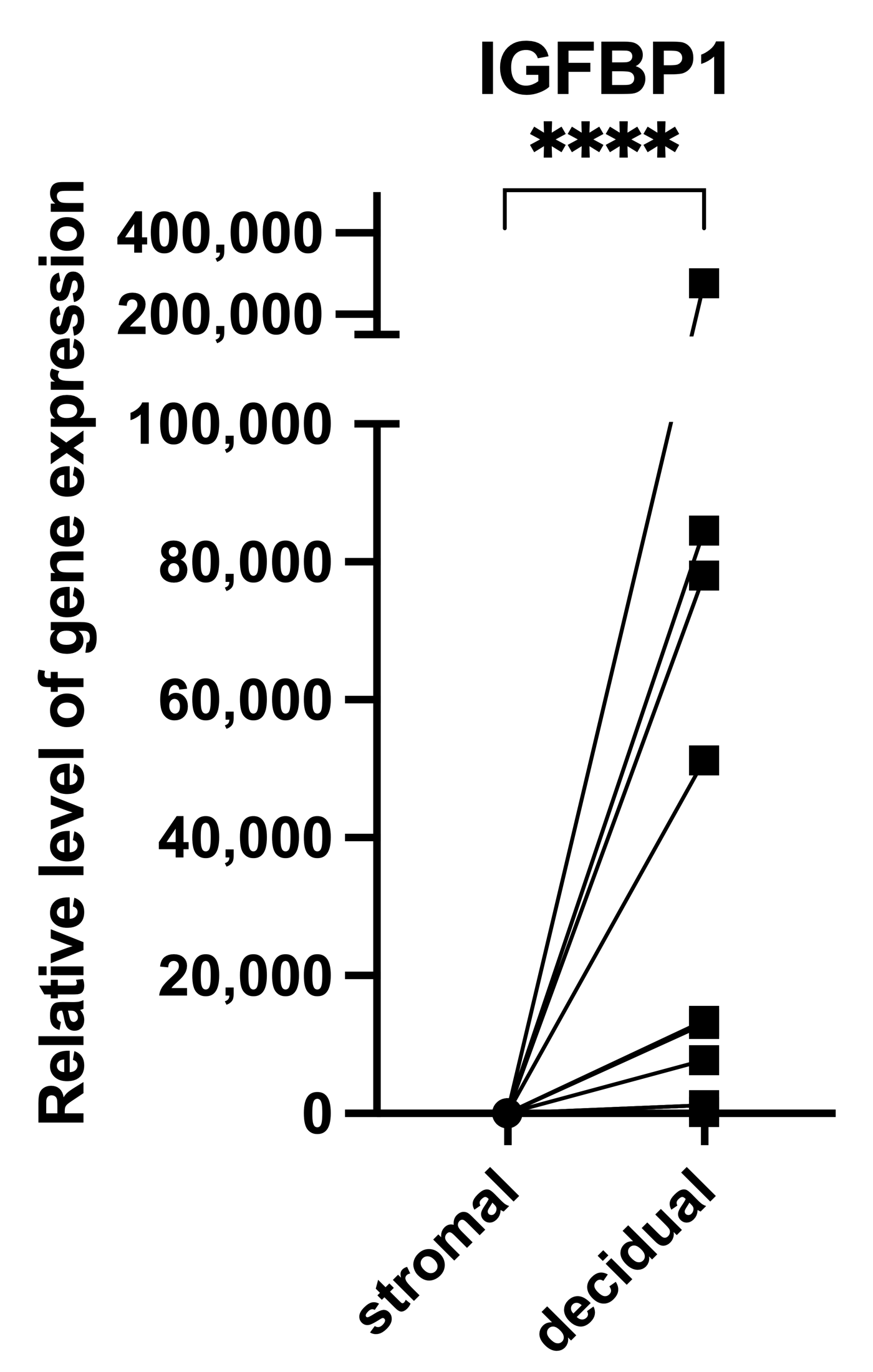

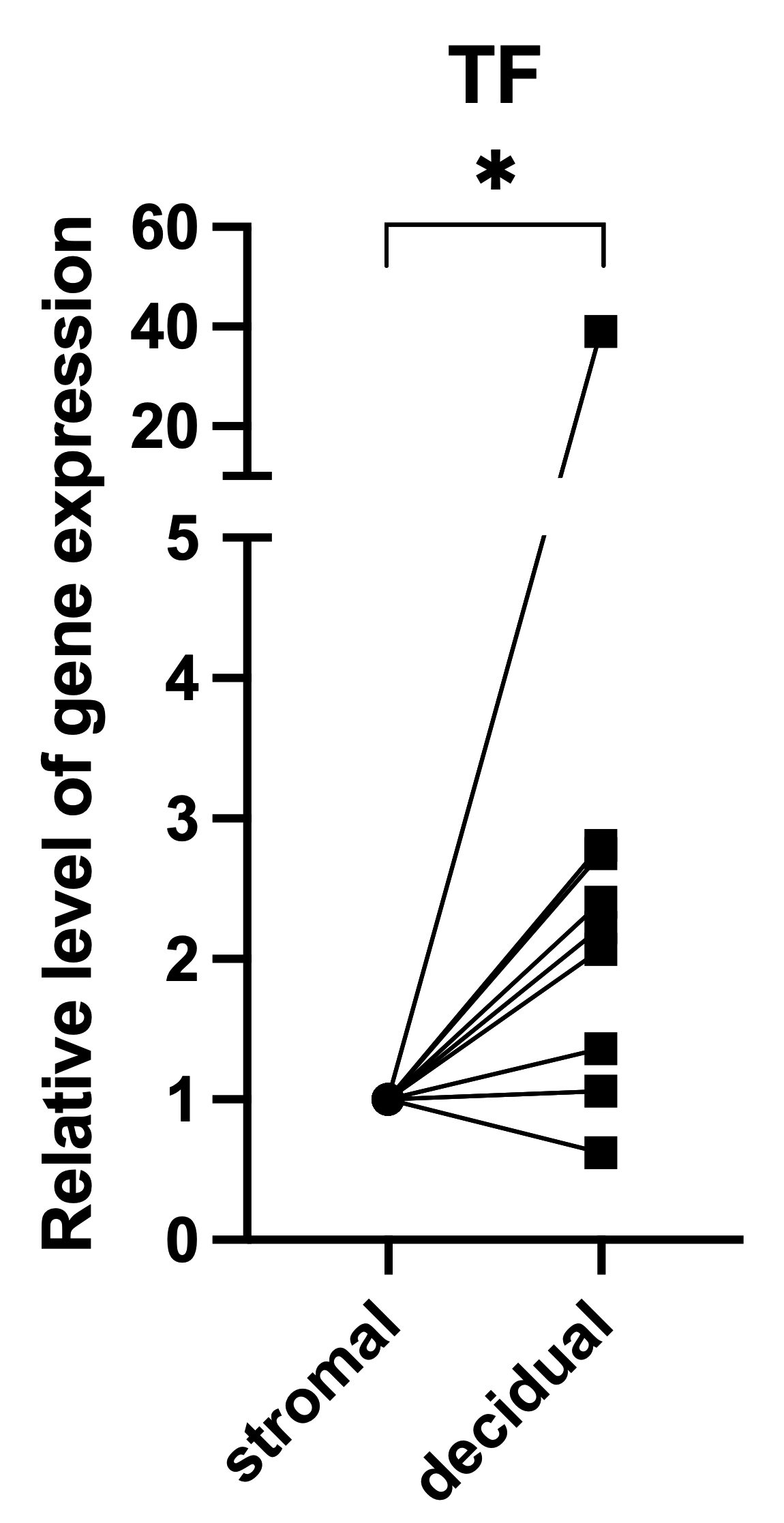

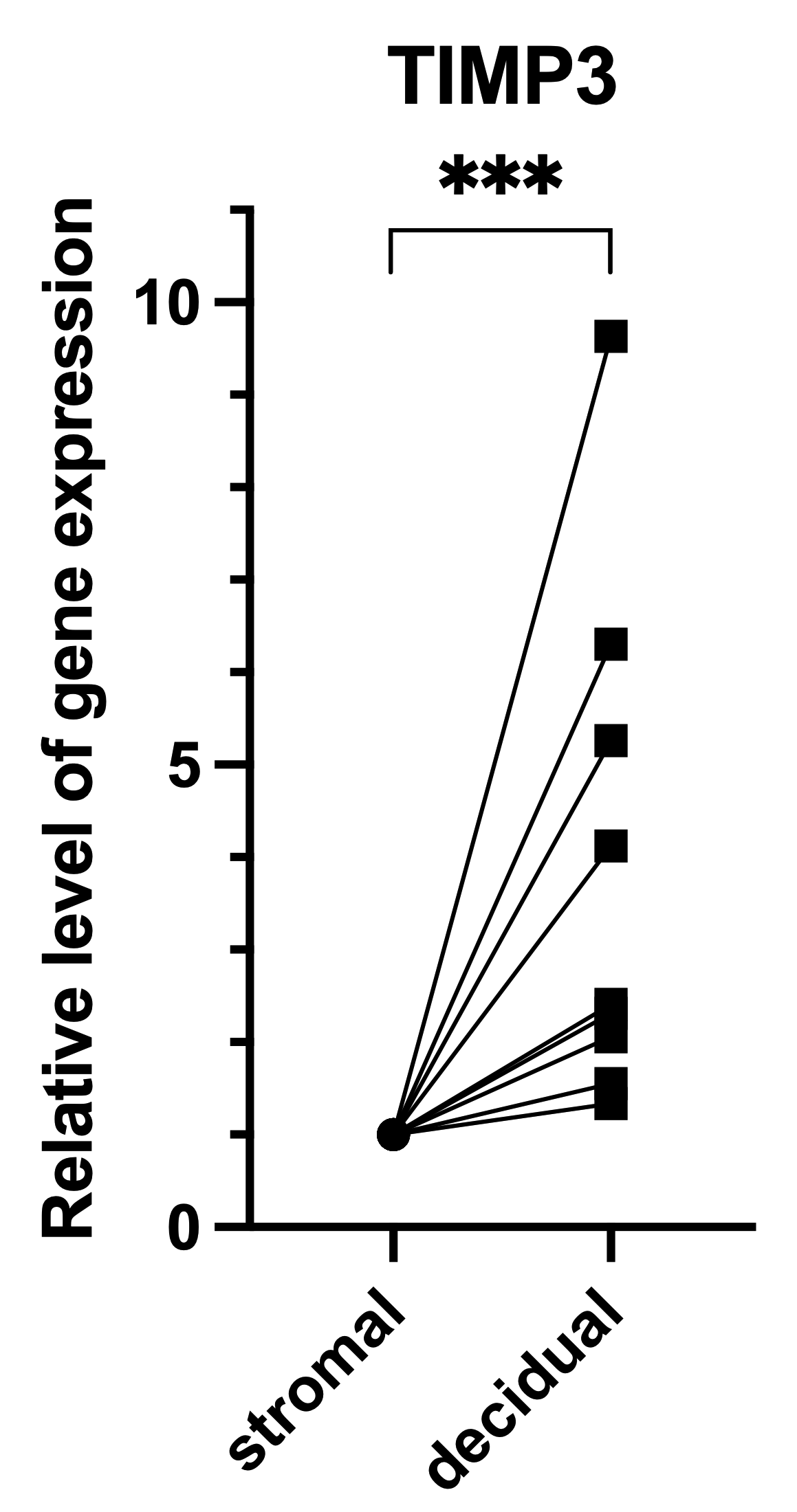

Supplement: Supplementary file 1 — Fig S1 [file JCMM-25-9523-s009.docx]

Supplementary figure 2

A C E


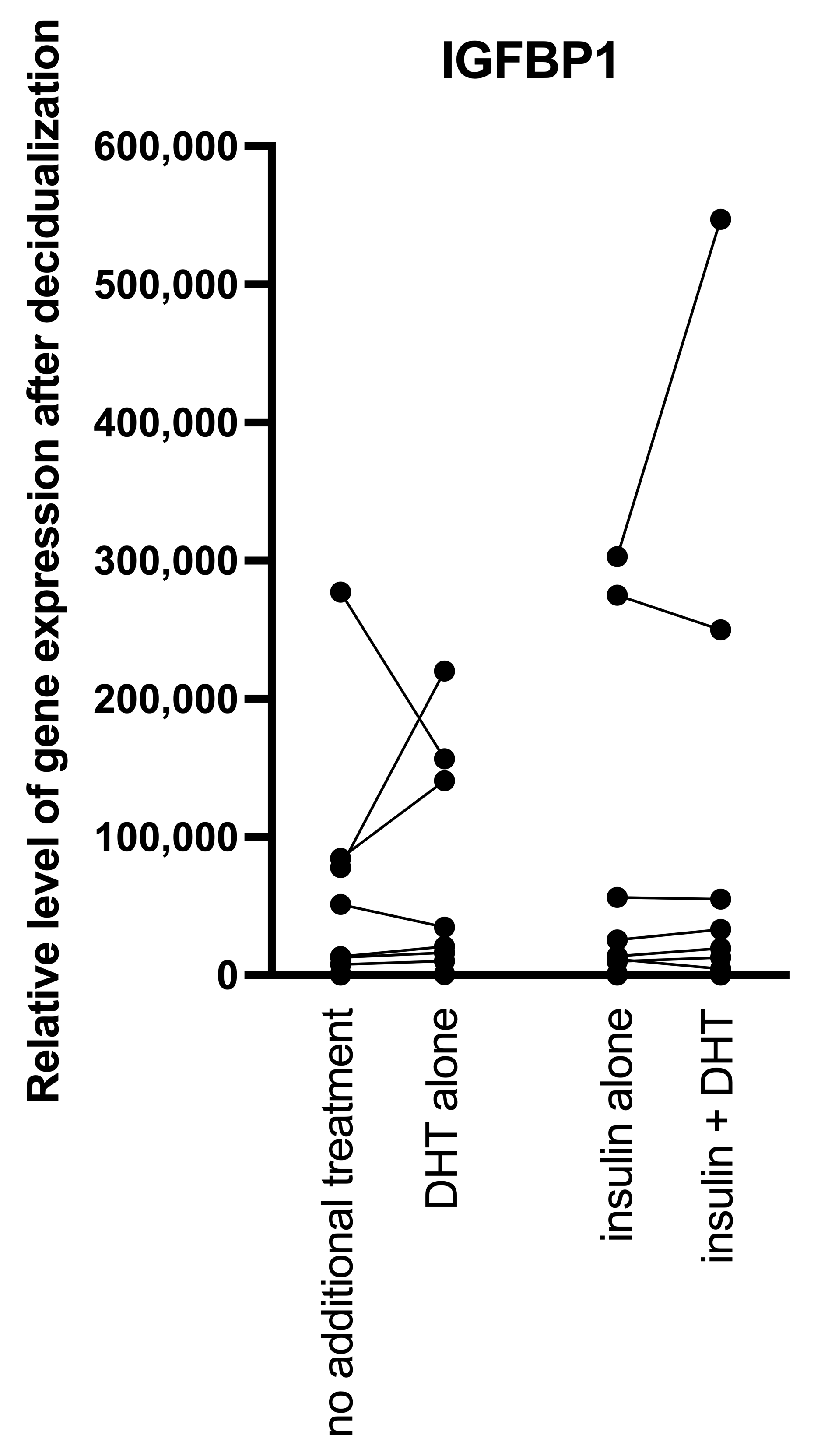

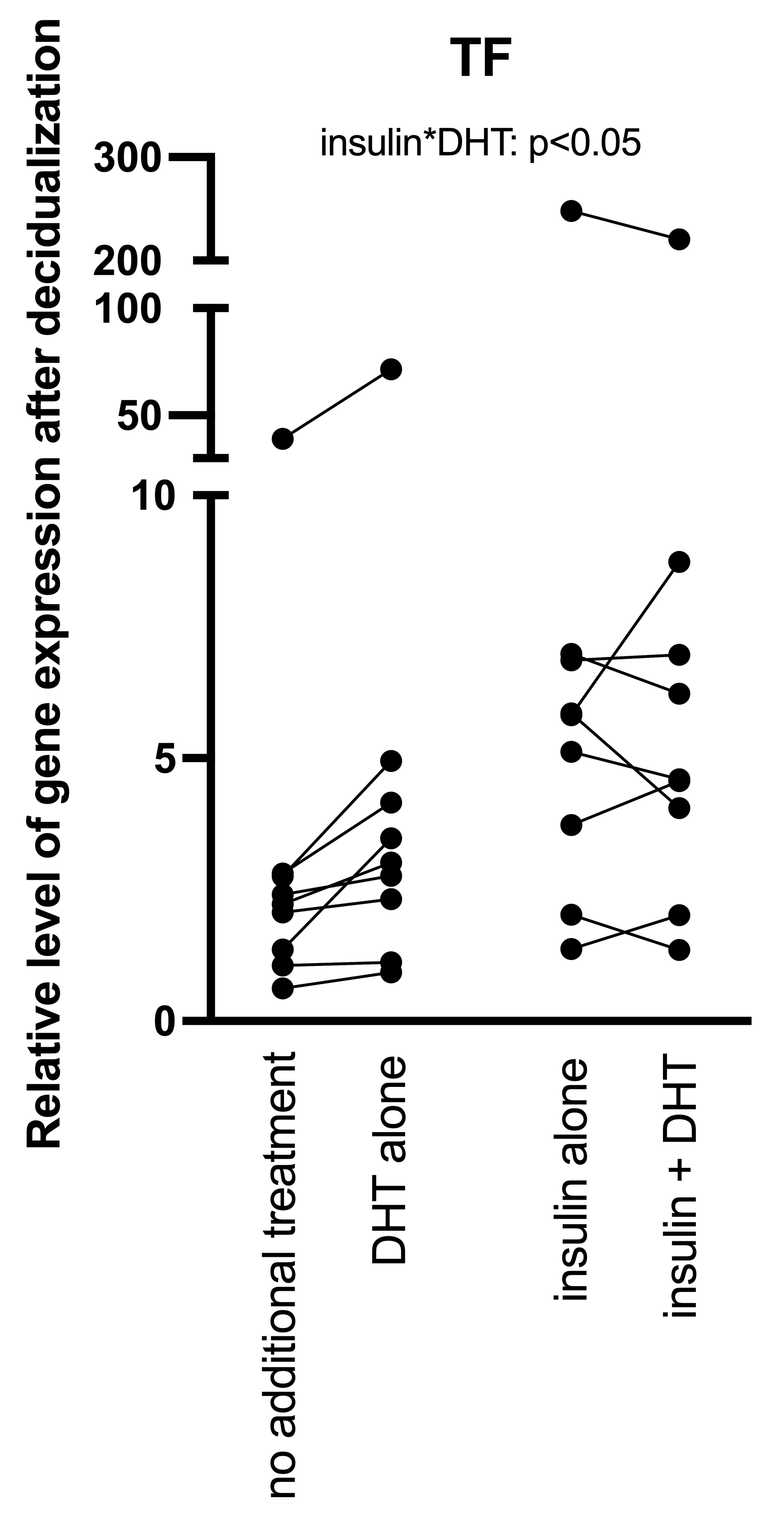

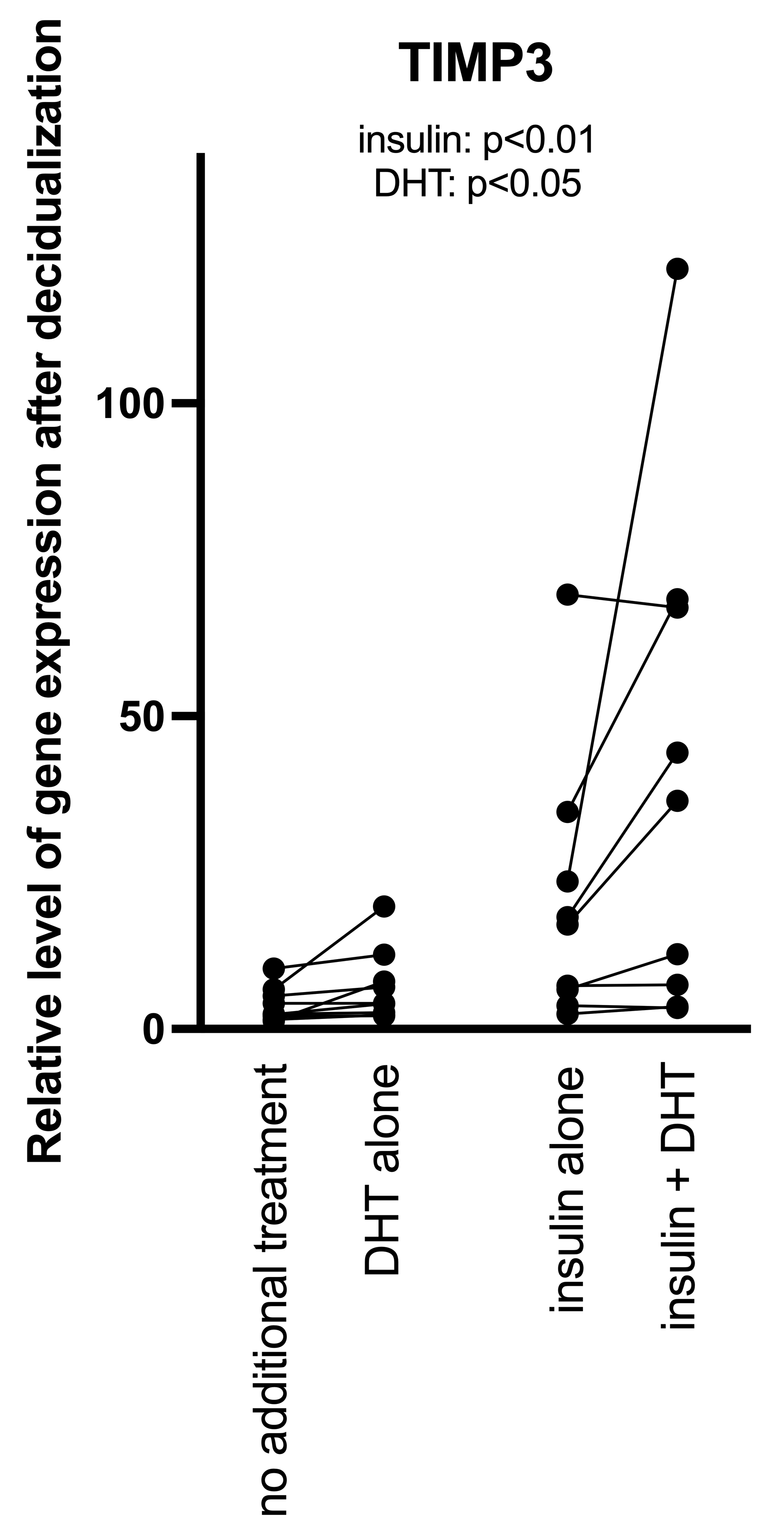


B D F


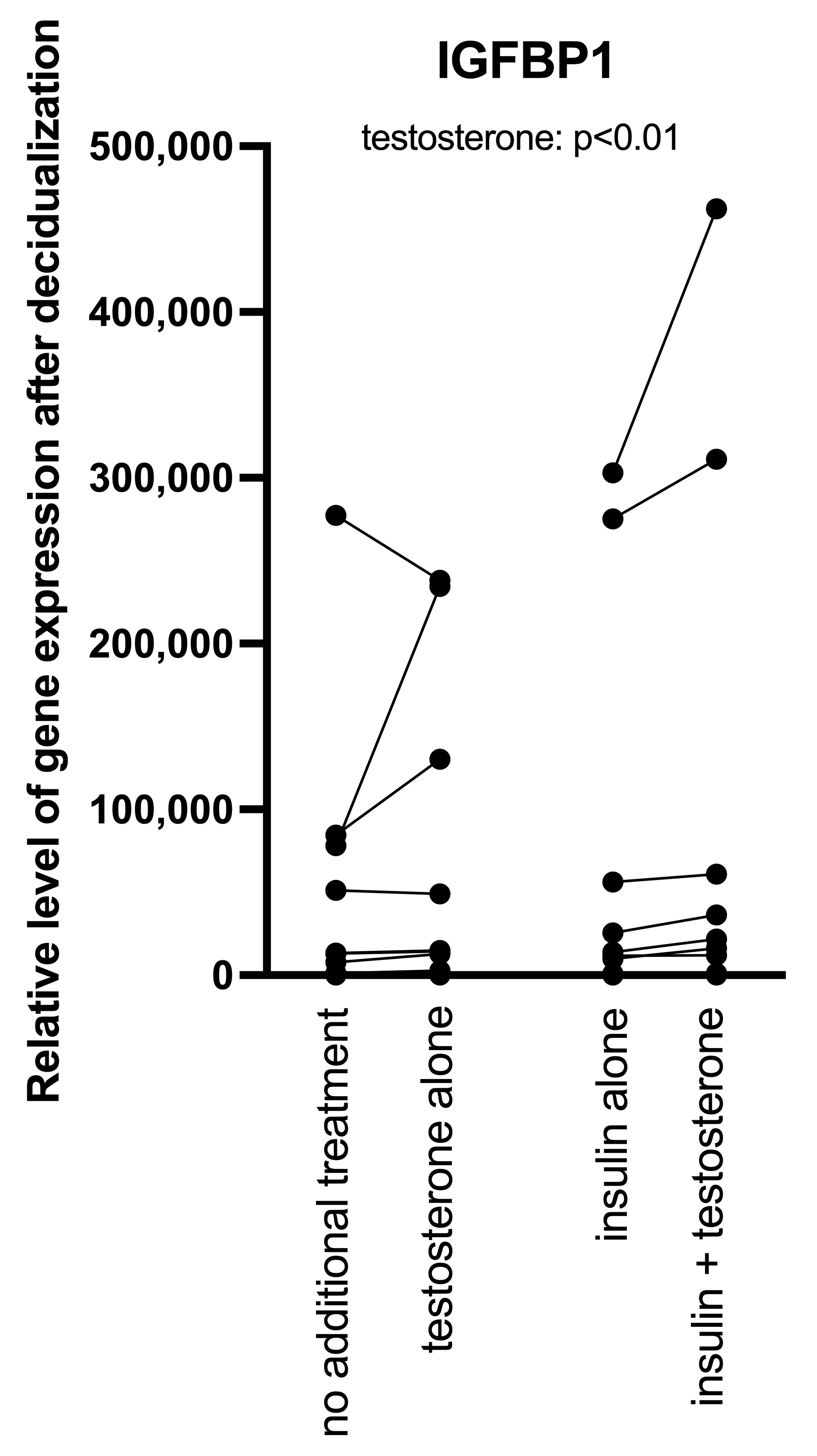

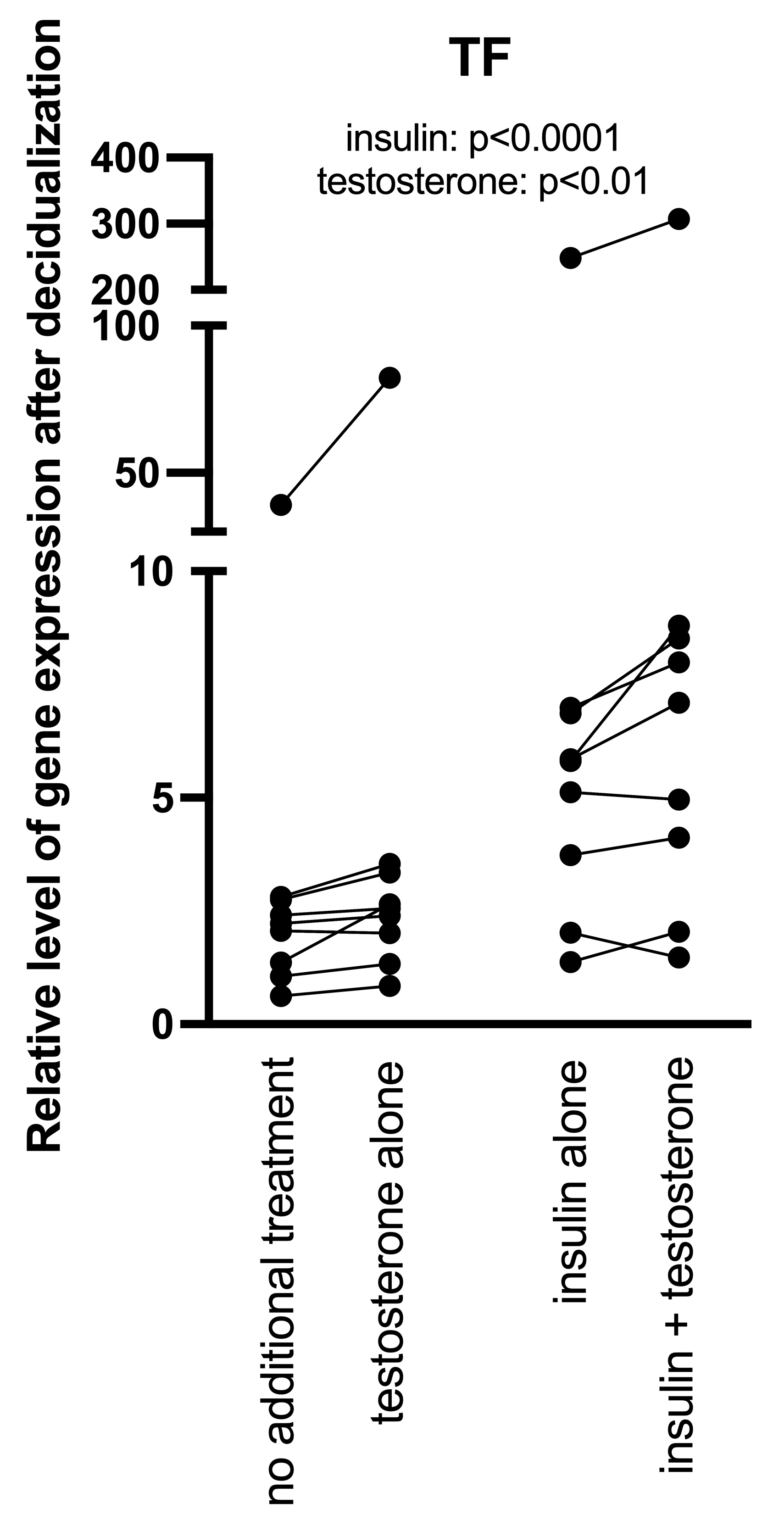

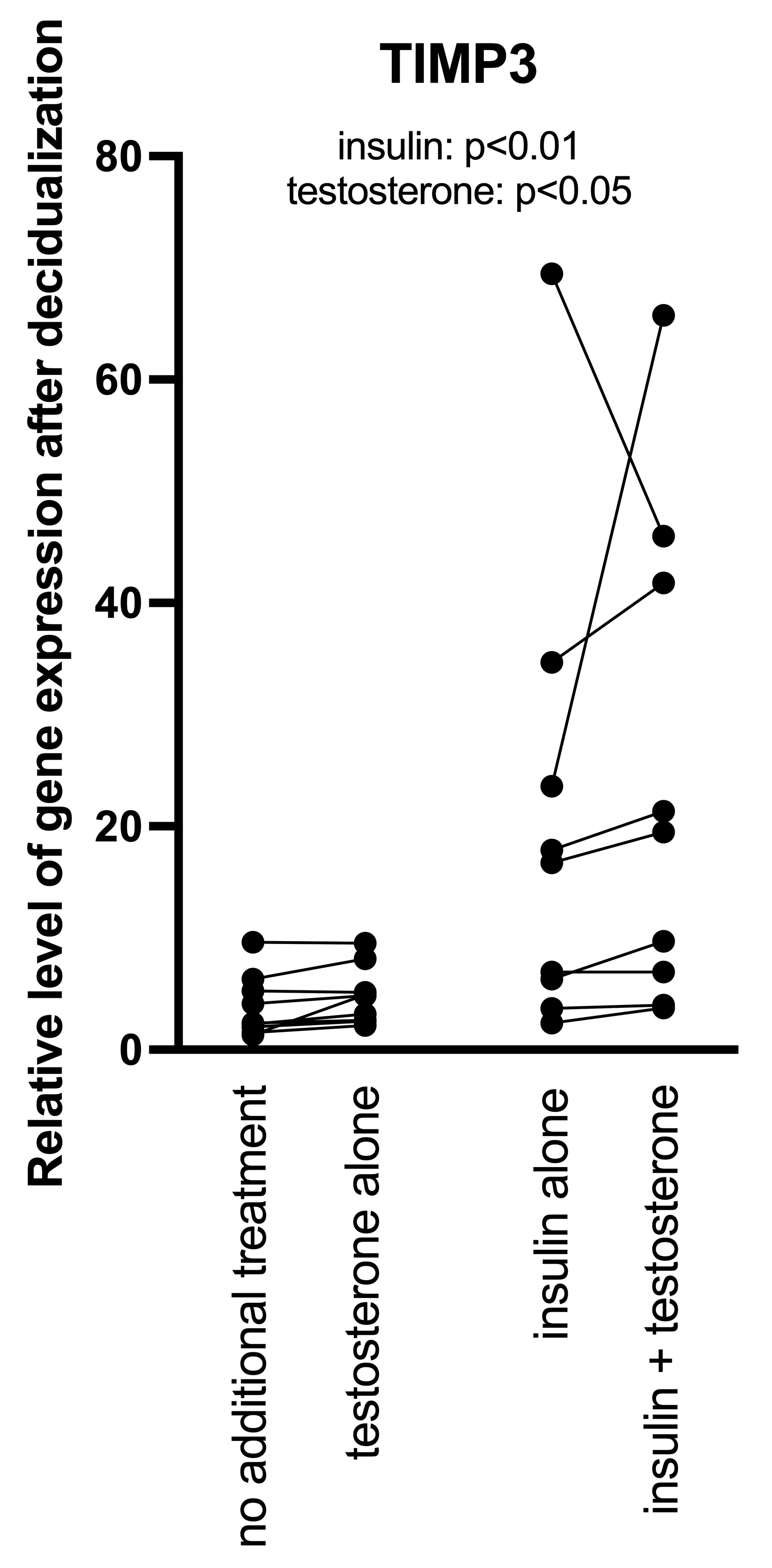

Supplement: Supplementary file 2 — Fig S2 [file JCMM-25-9523-s001.docx]

Supplementary figure 3


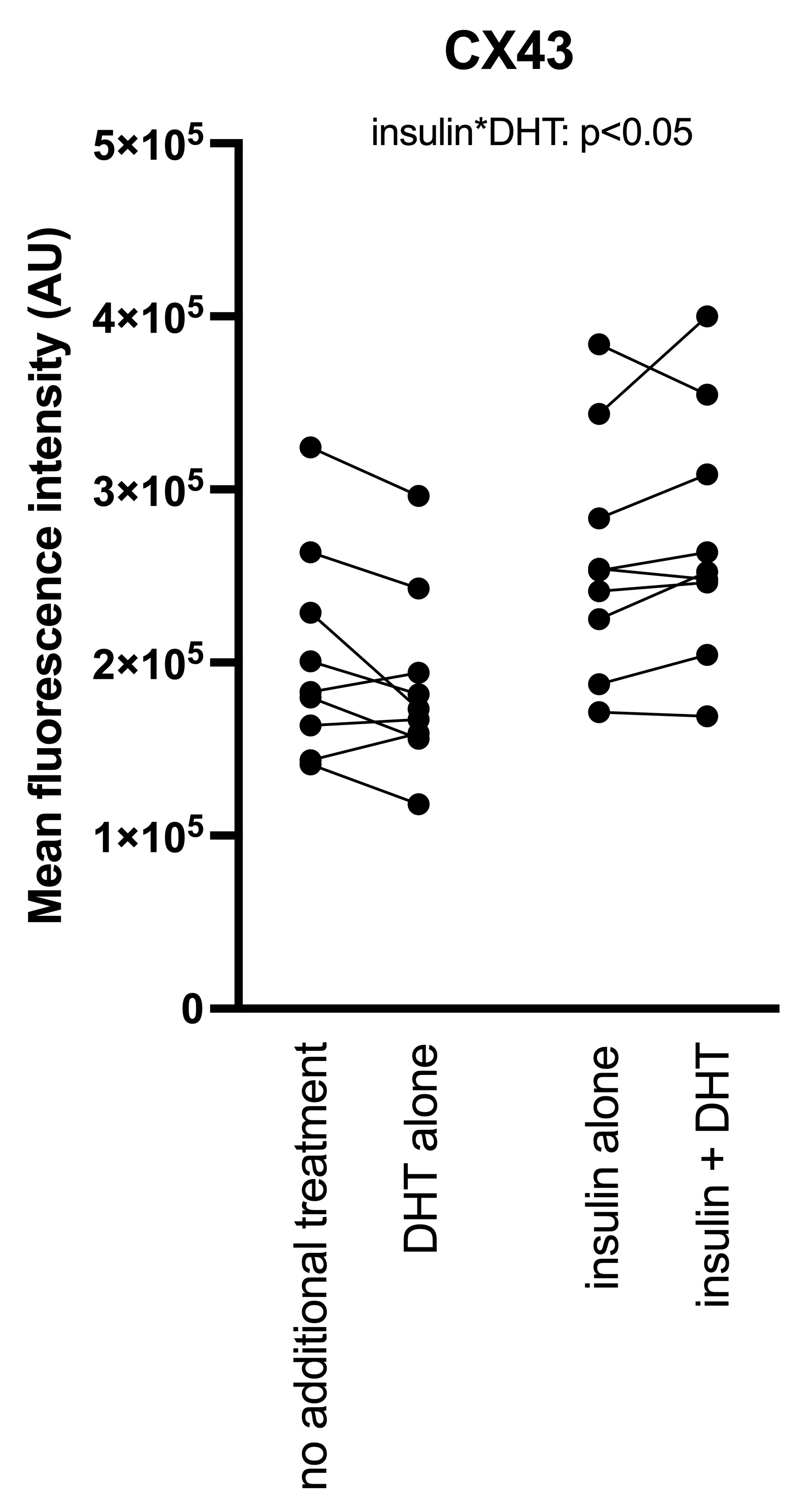

Supplement: Supplementary file 3 — Fig S3 [file JCMM-25-9523-s006.docx]

Supplementary figure 4

A B C D


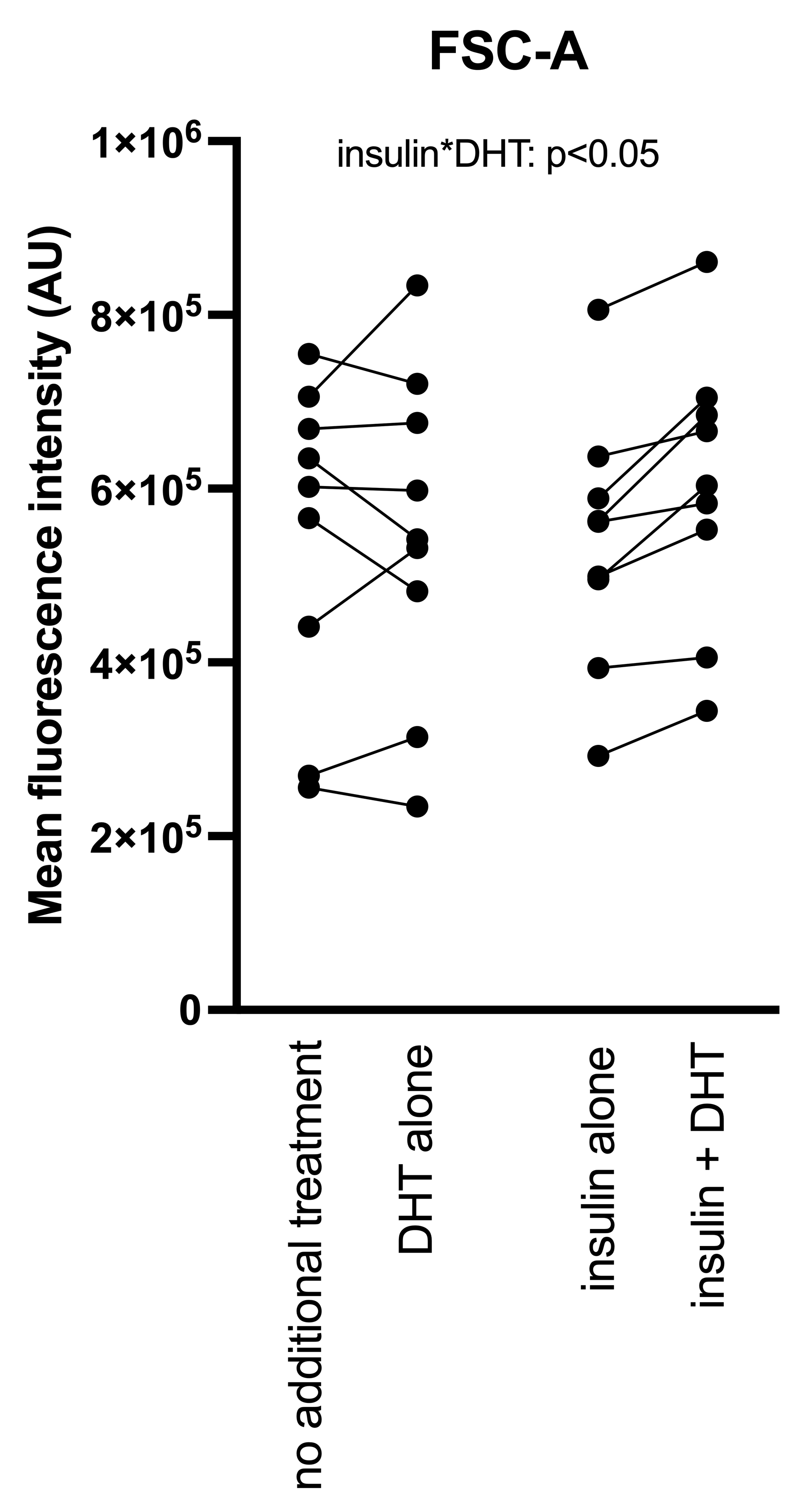

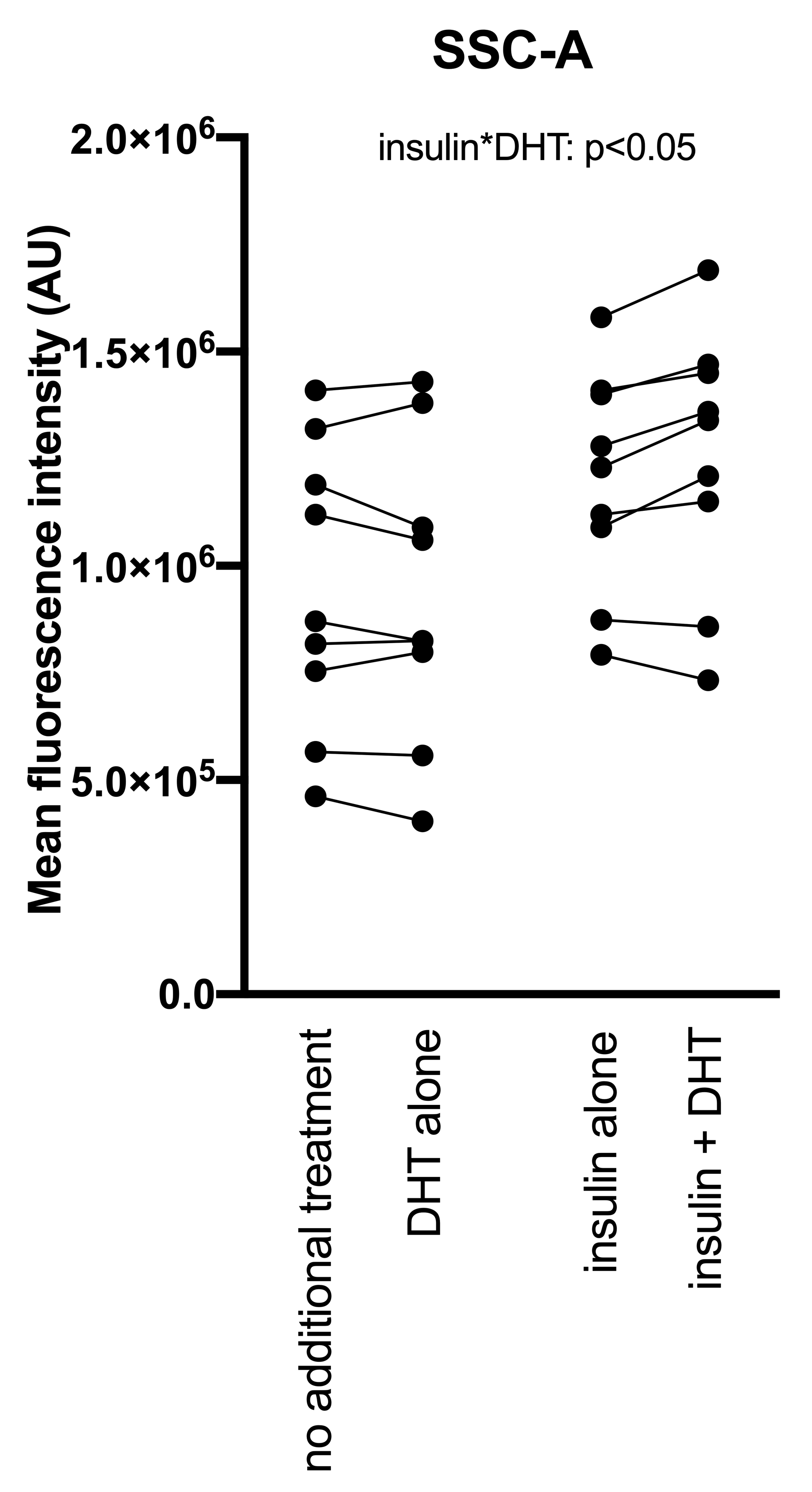

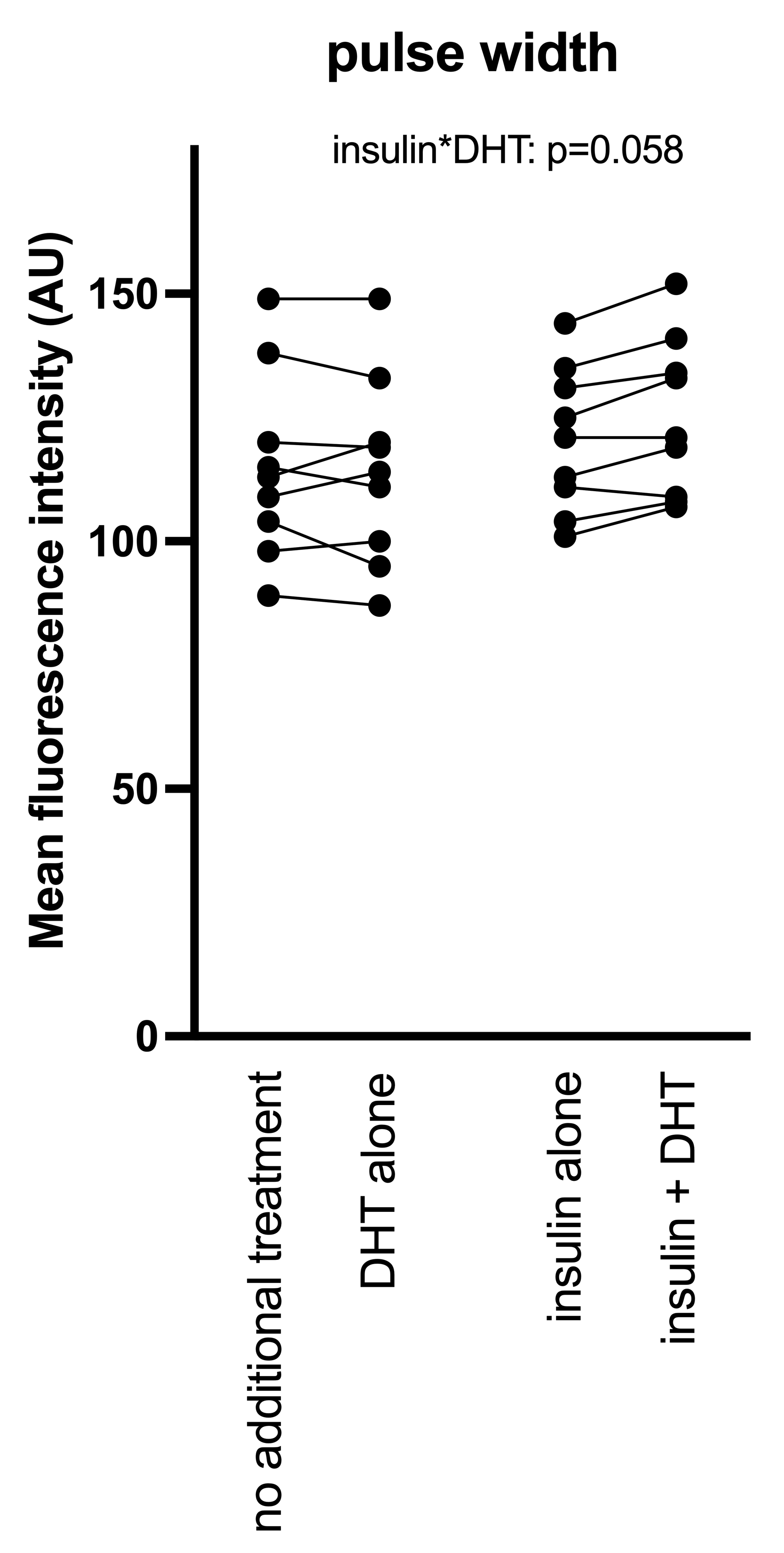

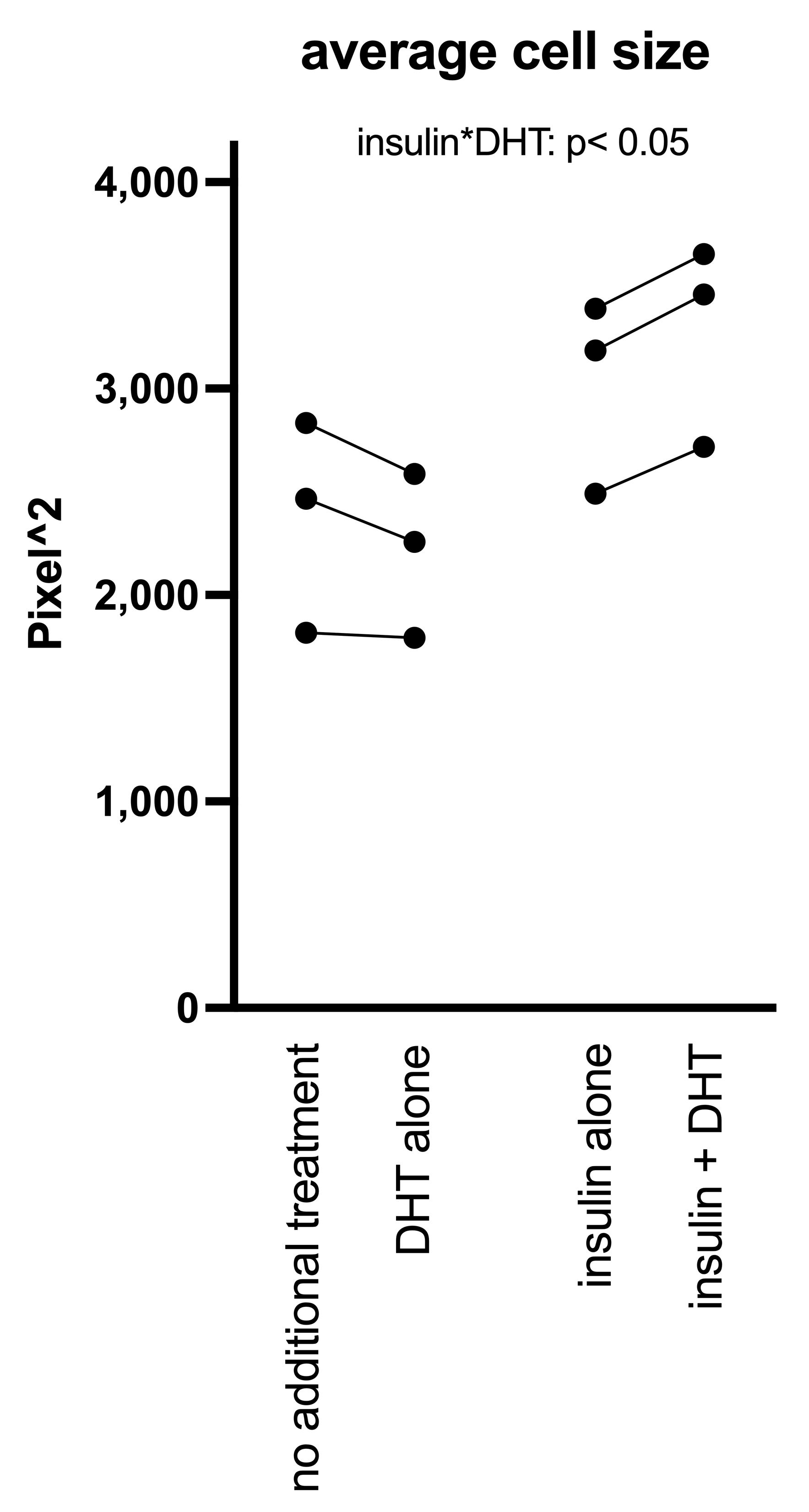

Supplement: Supplementary file 4 — Fig S4 [file JCMM-25-9523-s011.docx]
