# Supplementary material for: Interaction between insulin and androgen signalling in decidualization, cell migration and trophoblast invasion in vitro
Source: J Cell Mol Med. 2021 Aug 31;25(20):9523–32. doi: 10.1111/jcmm.16892 (PMC8505820; doi:10.1111/jcmm.16892)
Supplement: Supplementary file 5 — Table S1 [file JCMM-25-9523-s008.docx]

Supplementary table 1. TaqMan assays applied for amplification of PRL, IGFBP1 and RPL13A.

| Gene | TaqMan assay |
| --- | --- |
| PRL | Hs00168730_m1 |
| IGFBP1 | Hs00236877_m1 |
| RPL13A | Hs01926559_g1 |
