# Supplementary material for: Interaction between insulin and androgen signalling in decidualization, cell migration and trophoblast invasion in vitro
Source: J Cell Mol Med. 2021 Aug 31;25(20):9523–32. doi: 10.1111/jcmm.16892 (PMC8505820; doi:10.1111/jcmm.16892)
Supplement: Supplementary file 6 — Table S2 [file JCMM-25-9523-s012.docx]

Supplementary table 2. Forward and reverse oligos applied for amplification of TF, TIMP3 and RPL13A.

| Gene | Sequence |
| --- | --- |
| TF | 5` -CCA AAC CCG TCA ATC AAG TC- 3` |
|  | 5` -CGT CTG CTT CAC ATC CTT CA- 3` |
| TIMP3 | 5` -GGG GAA GAA GCT GGT AAA GG- 3` |
|  | 5` -ATC TTG GTG AAG CCT CGG TA- 3` |
| RPL13A | 5` -CAG GTC CTG GTG CTT GAT G - 3` |
|  | 5` - GTT GAT GCC TTC ACA GCG TA- 3` |
