# Supplementary material for: Interaction between insulin and androgen signalling in decidualization, cell migration and trophoblast invasion in vitro
Source: J Cell Mol Med. 2021 Aug 31;25(20):9523–32. doi: 10.1111/jcmm.16892 (PMC8505820; doi:10.1111/jcmm.16892)
Supplement: Supplementary file 7 — Table S3 [file JCMM-25-9523-s003.docx]

Supplementary table 3. Closure of the wound of *in vitro* decidualized endometrial stromal cells and HTR-8/SVneo spheroid invasion in co-culture with *in vitro* decidualized endometrial stromal cells in response to insulin, DHT and insulin+DHT

| **Measure** | **Decidual** | **Insulin** | **DHT** | **Insulin+DHT** | **Interaction**  **and**  **Main effect** | **Post-hoc effects** |
| --- | --- | --- | --- | --- | --- | --- |
| Closure of the wound (%) | 57.56 (45.12-74.96) | 45.72 (42.63-63.35) | 57.09 (52.96-70.21) | 39.29 (27.17-41.92) | 1^(p=0.102)^ | b^(**)^, d^(*)^ |
| Spheroid invasion 12 h | 3.29 (2.15-5.10) | 2.68 (2.13-3.66) | 3.14 (2.36-5.23) | 1.87 (1.38-3.24) | 1^(p=0.111)^ | b^(*)^, d^(*)^ |
| Spheroid invasion 16 h | 4.93 (3.89-6.68) | 3.36 (3.12-4.63) | 4.93 (3.88-6.99) | 2.74 (2.23-4.28) | A^(**)^ |  |

Data are median and ranges (min-max). Values of spheroid invasion are fold changes compared with the area of the spheroids at 0h in each condition. Effect: 1 = Interaction between insulin and DHT; A = Main effect of insulin. Post-Hoc test: a = insulin vs. decidual, b = insulin+DHT vs. DHT, c = DHT vs. decidual, d = insulin+DHT vs. insulin. *= p < 0.05 and **= p < 0.01.
