# Supplementary material for: Interaction between insulin and androgen signalling in decidualization, cell migration and trophoblast invasion in vitro
Source: J Cell Mol Med. 2021 Aug 31;25(20):9523–32. doi: 10.1111/jcmm.16892 (PMC8505820; doi:10.1111/jcmm.16892)
Supplement: Supplementary file 12 — Supplementary Material [file JCMM-25-9523-s002.docx]

**Supplementary figure legends**

**Supplementary figure 1**

Relative levels of PRL (A), IGFBP1 (B), TF (C) and TIMP3 (D) gene expressions in stromal cells (closed circles) and in *in vitro* decidualized (treated with MPA and db-cAMP) stromal cells (closed squares) of healthy volunteers after 6 days. *p<0.05; ***p<0.001; ****p<0.0001

**Supplementary figure 2**

Relative levels of IGFBP1 (A), TF (C) and TIMP3 (E) gene expressions after 6 days in response to insulin and/or DHT in *in vitro* decidualized (treated with MPA and db-cAMP) human endometrial stromal cells of healthy volunteers. Interaction effect on TF gene expression between insulin and DHT: p<0.05. Main effect of insulin on TIMP3 gene expression: p<0.01; main effect of DHT on TIMP3 gene expression: p<0.05.

Relative levels of IGFBP1 (B), TF (D) and TIMP3 (F) gene expressions after 6 days in response to insulin and/or testosterone in *in vitro* decidualized (treated with MPA and db-cAMP) human endometrial stromal cells of healthy volunteers. Main effect of insulin on TF gene expression: p<0.0001; on TIMP3 gene expression: p<0.01; main effect of testosterone on IGFBP1 gene expression: p<0.01; on TF gene expression: p<0.01; on TIMP3 gene expression: p<0.05.

**Supplementary figure 3**

Mean fluorescence intensity of CX43 after 6 days in response to insulin and/or DHT in *in vitro* decidualized (treated with MPA and db-cAMP) human endometrial stromal cells of healthy volunteers. Interaction effect between insulin and DHT: p<0.05.

**Supplementary figure 4**

Mean fluorescence intensity of FSC-A (A), SSC-A (B), and pulse width (C) and average cell size (D) observed using light microscopy and measured with ImageJ in response to insulin and/or DHT in *in vitro* decidualized (treated with MPA and db-cAMP) human endometrial stromal cells of healthy volunteers after 6 (FSC-A, SSC-A, pulse width) and 5 days (average cell size), respectively. Interaction effect between insulin and DHT on FSC-A: p<0.05; on SSC-A: p<0.05; on pulse width: p=0.058; on average cell size: p<0.05.

**Supplementary video 1**

Co-culture invasion assay using HTR-8/SVneo trophoblast spheroid and endometrial stromal cells decidualized (treated with MPA and db-cAMP) for 6 days.

**Supplementary video 2**

Co-culture invasion assay using HTR-8/SVneo trophoblast spheroid and endometrial stromal cells decidualized (treated with MPA and db-cAMP) and treated with 100 nM insulin for 6 days.

**Supplementary video 3**

Co-culture invasion assay using HTR-8/SVneo trophoblast spheroid and endometrial stromal cells decidualized (treated with MPA and db-cAMP) and treated with 1 μM DHT for 6 days.

**Supplementary video 4**

Co-culture invasion assay using HTR-8/SVneo trophoblast spheroid and endometrial stromal cells decidualized (treated with MPA and db-cAMP) and treated with 100 nM insulin and 1 μM DHT for 6 days.
